# Gene expression in human brain implicates sexually dimorphic pathways in autism spectrum disorders

**DOI:** 10.1038/ncomms10717

**Published:** 2016-02-19

**Authors:** Donna M. Werling, Neelroop N. Parikshak, Daniel H. Geschwind

**Affiliations:** 1 Center for Neurobehavioral Genetics, Semel Institute, David Geffen School of Medicine, University of California, Los Angeles, Los Angeles, California 90095, USA; 2 Program in Neurogenetics, Department of Neurology, David Geffen School of Medicine, University of California, Los Angeles, Los Angeles, California 90095, USA; 3 Center for Autism Research and Treatment, Semel Institute, David Geffen School of Medicine, University of California, Los Angeles, Los Angeles, California 90095, USA; 4 Department of Human Genetics, David Geffen School of Medicine, University of California, Los Angeles, Los Angeles, California 90095, USA

## Abstract

Autism spectrum disorder (ASD) is more prevalent in males, and the mechanisms behind this sex-differential risk are not fully understood. Two competing, but not mutually exclusive, hypotheses are that ASD risk genes are sex-differentially regulated, or alternatively, that they interact with characteristic sexually dimorphic pathways. Here we characterized sexually dimorphic gene expression in multiple data sets from neurotypical adult and prenatal human neocortical tissue, and evaluated ASD risk genes for evidence of sex-biased expression. We find no evidence for systematic sex-differential expression of ASD risk genes. Instead, we observe that genes expressed at higher levels in males are significantly enriched for genes upregulated in post-mortem autistic brain, including astrocyte and microglia markers. This suggests that it is not sex-differential regulation of ASD risk genes, but rather naturally occurring sexually dimorphic processes, potentially including neuron–glial interactions, that modulate the impact of risk variants and contribute to the sex-skewed prevalence of ASD.

## Abstract (web summary)

Autism spectrum disorder is approximately 4.5 times more likely to occur in boys than girls. Here, Werling, Geschwind and Parikshak characterized sexually dimorphic gene expression in the non-diseased, post-mortem, adult and prenatal human brain, and show genes expressed at higher levels in males are significantly enriched for genes upregulated in autistic brain.

## Graphical abstract

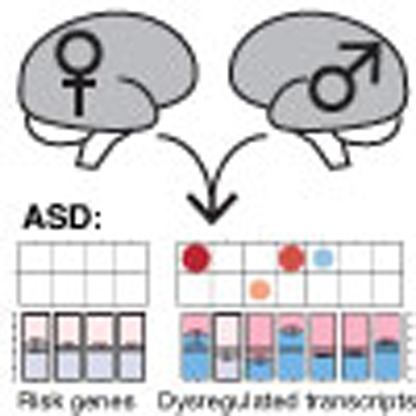

Autism spectrum disorder (ASD) is a developmental condition characterized by deficits in social communication and restricted, repetitive behaviours or interests^1^ that is estimated to affect 1 in 68 children in the United States^2^. Genetic variation contributes strongly to ASD risk, as evidenced by high familial recurrence^3^,^4^,^5^, overlap with monogenic syndromes such as Fragile X (*FMR1*) or Smith–Lemli–Opitz syndrome (*DHCR7*)^6^, and higher rates of gene-disrupting genetic variants in ASD cases compared with their siblings or controls^7^,^8^,^9^. The set of genes that can be definitively implicated as ASD risk genes has been growing rapidly, and predictive models from studies of single nucleotide variants in sporadic cases estimate that there are likely to be hundreds of genes involved in ASD risk^8^,^10^. Given the scope of this genetic heterogeneity, understanding the biological aetiologies of ASD and designing broadly applicable treatments has proven challenging.

One robust risk factor for ASD is sex: for every female with ASD in the United States, there are 4.5 affected males^2^, and a male bias in prevalence is consistent across countries and across diagnostic criteria^11^. A multiple threshold liability model has been applied to conceptualize this difference in vulnerability, which posits that a higher minimum liability is required for females to manifest the ASD phenotype as compared with males^12^,^13^,^14^; this is also referred to as the female protective model. There is now evidence from population-wide data, family-level data^14^ and at the genetic level^7^,^9^,^15^ to support the hypothesis from this model that autistic females with ASD carry greater genetic liability. However, we note that a female protective model is not incompatible with the existence of male-specific risk factors, and the molecular mechanisms responsible for either protecting females or potentiating males' vulnerability remain unknown.

Several theories have been proposed regarding sex-differential risk and protective mechanisms, though the evidence supporting each is varied^12^. For example, ASD has been conceptualized as an X-linked disorder^16^,^17^, and although several X-chromosome genes have been implicated, numerous autosomal genes contribute to risk as well^7^,^8^; X-chromosome genes alone do not explain the overall male bias^9^,^18^,^19^. Imprinted, paternally expressed X-chromosome genes have also been proposed to exert protective effects on females^20^, though such genes have yet to be identified. Beyond genetics, the extreme male brain theory of autism posits that elevated prenatal testosterone exposure increases risk^21^. While recent evidence has linked increased fetal testosterone levels to later ASD diagnoses in a population sample^22^, the molecular and cellular mechanisms that act to link an early hormone exposure to a later ASD phenotype are unknown. It is also plausible that multiple mechanisms, acting independently or interacting with one another, collectively account for the sex bias in ASD risk.

We reasoned that since sex-differential gene expression patterns contribute to the development and function of a sexually dimorphic brain, evaluating genome-wide sex-differential gene expression in neural tissue could elucidate points of overlap with ASD risk genes and related pathways. Here we test two basic hypotheses about the relationship between sexually dimorphic expression and ASD risk genes: (1) ASD risk genes are expressed at different levels in males and females. With differing baseline expression levels, the magnitude of the impact of a disruptive mutation in a risk gene is likely to differ by sex. Accordingly, we expect to observe enrichment of sex-differentially expressed (sex-DE) genes among known ASD risk genes. (2) ASD risk genes are expressed at the same level in males and females, but genes in interacting molecular pathways and/or cellular processes are differentially expressed by sex. In this case, the downstream impact of sex-neutrally expressed ASD risk genes is likely modulated by their interactions with sexually dimorphic processes. If this were the case, we would expect to observe enrichment of sex-DE genes among gene sets representing processes associated with ASD pathophysiology, but not among ASD risk genes themselves.

To evaluate these hypotheses, we identify sexually dimorphic gene expression patterns in adult and prenatal human cerebral cortex tissue and test ASD-associated gene sets for enrichment of sex-differential expression. We observe significantly male-biased expression patterns for microglial-function-enriched, ASD-upregulated, co-expression modules from post-mortem ASD brain^23^,^24^ and microglia and astrocyte marker genes^25^,^26^,^27^, but no enrichment of sex-differential expression for ASD risk genes. These patterns are most consistent with our second hypothesis, and suggest that sex differences in biological processes captured by specific ASD-dysregulated co-expression modules, or sexual dimorphisms in cortical microglia and/or astrocytes, may play critical roles in setting males' greater risk for ASD.

## Results

To identify sex-differential gene expression patterns in the human brain, we analysed RNA-sequencing (RNA-seq) gene expression data from human cerebral cortex tissue from the BrainSpan project^28^ and from an independent, in-house data set, as well as published array data from prenatal samples^29^. We focused on cerebral cortex, since ASD risk genes are highly expressed in this region in neurotypical brain^30^,^31^, and examination of post-mortem autistic brain has demonstrated consistent gene expression changes in the cerebral cortex as well^23^,^24^. To characterize stable sex differences in gene expression, we evaluated samples from adult subjects, and to assess sex-differential expression during early development, when many ASD risk genes are highly expressed, we evaluated samples from prenatal subjects. We then tested multiple ASD risk gene sets, ASD-associated gene sets and cell type markers for enrichment of sex-differential expression^8^,^23^,^24^,^25^,^26^,^27^,^32^,^33^,^34^, to determine whether sexually dimorphic utilization of ASD risk genes, or genes in interacting processes, may contribute to the sex bias in ASD prevalence.

### Sex-differential expression in adult human cortex

We first analysed RNA-seq data from 58 post-mortem cortex samples from the BrainSpan project^28^ for sex-differential gene expression using a linear mixed model. This set of samples was collected from 10 teenage and adult subjects matched for sex, age and brain region (13–40 years; 5 females), and filtered for outliers (Supplementary Table 1).

**Figure 1:**
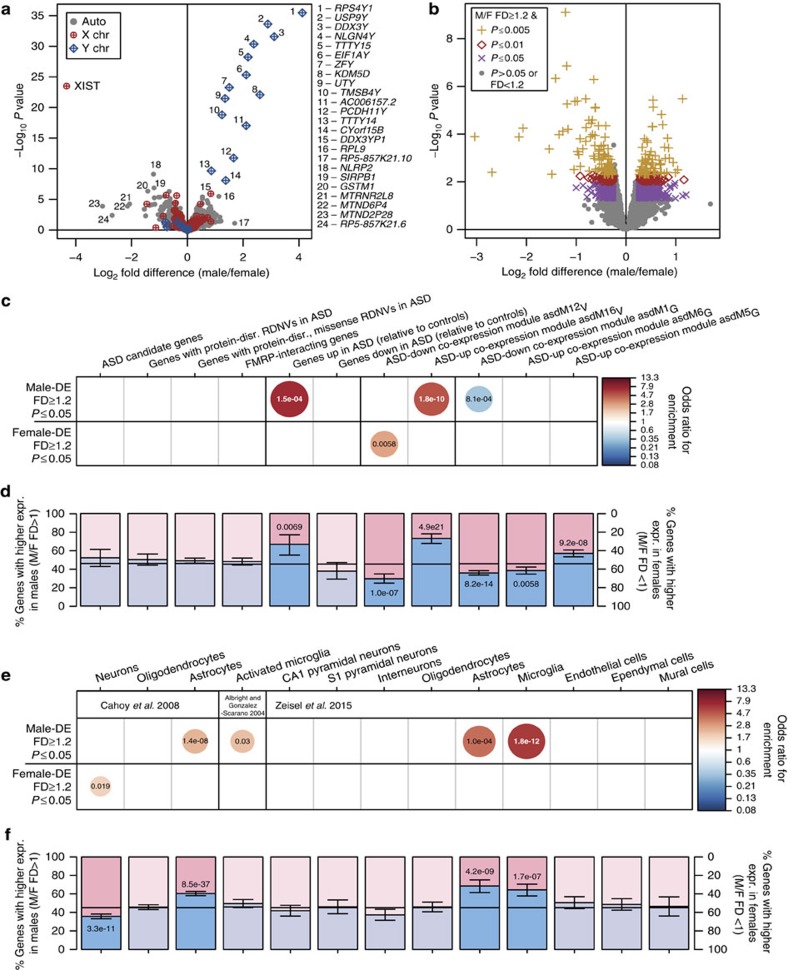
Microglia and astrocyte markers and genes upregulated in ASD brain tend toward higher expression in adult male brain. (**a**) Volcano plot for the differential expression results from all 16,392 transcripts expressed in the adult BrainSpan sample (*n* male=29 samples from 5 subjects, *n* female=29 samples from 5 subjects). (**b**) Subset of the volcano plot in **a** for all 15,724 autosomal transcripts. (**c**) Enrichment for ASD risk genes and ASD-associated gene expression patterns, and (**e**) neural cell type markers, within male-DE (higher expression in males, FD⩾1.2, *P*⩽0.05; 439 genes) and female-DE (higher expression in females, FD⩾1.2, *P*⩽0.05; 427 genes) gene sets by Fisher's exact test; circle size and colour indicate the odds ratio of all significant overlaps (Bonferroni-adjusted *P* value <0.05); overlaid text displays the adjusted *P* value for each enrichment. (**d**) Shifts in the distribution of sex-differential expression direction for genes in each ASD risk or ASD expression sets, and (**f**) neural cell type markers. Blue and pink bars display the proportions of each gene set that have higher expression in males (FD>1) or females (FD<1); whiskers note 95% confidence intervals; horizontal black lines note the proportion of male- and female-higher genes in the corresponding background gene set. Overlaid text displays significant Bonferroni-adjusted *P* values from the binomial test. chr, chromosome; disr., disrupting; expr., expression; F, female; M, male.

Sex-differential expression patterns in adult cortex show expected, robust differential expression of Y-chromosome genes and *XIST*, an X-chromosome transcript that initiates X-chromosome inactivation and is only expressed in females (Fig. 1a). We note that all Y-chromosomal transcripts that fail to show male-biased expression are pseudogenes with high sequence similarity to their corresponding genes on other chromosomes. Previous studies of sex-differential expression in human brain have preferentially reported sex-DE genes with pronounced fold differences (FDs), such as those on the X and Y chromosomes^29^,^35^,^36^. Beyond these differences driven by sex chromosome copy number, sex-differential expression of autosomal and non-*XIST* X-chromosome transcripts are generally subtle in magnitude^37^, and may reflect sex-specific tuning of molecular pathways. Using a minimum FD magnitude of 1.2, a standard threshold, we identify 186 genes at *P*<0.005, 311 genes at *P*<0.01 and 866 genes at *P*<0.05 (Fig. 1b and Supplementary Data 1); just 58 genes are sex-DE at a Benjamini–Hochberg adjusted *P* value of 0.05, 21 of which are on the sex chromosomes. The 37 autosomal sex-DE genes, then, are not sexually dimorphic due to copy number differences between males and females. Instead, they may be the regulatory targets of sex steroid hormone receptors or transcription factors from the X or Y chromosomes, or they may be preferentially expressed in a certain cell type that is differentially represented in male and female cortex.

### Over-representation analysis for gene sets of interest

To determine whether genes associated with ASD risk show sex-differential expression in the human adult cortex, we examined sets of known risk genes from several sources: (a) candidate genes from a manually curated database^32^; (b) genes with rare, *de novo*, protein-disrupting or missense single nucleotide variants in sporadic ASD cases from the Simons Simplex Collection^8^; and (c) FMRP (Fragile X mental retardation protein) binding targets^33^ (Supplementary Data 2). Together, these gene sets capture a broad scope of ASD risk genes, including heavily studied candidates, genes with evidence of *de novo* risk variants, and genes from a strongly ASD-associated regulatory network^33^. For each gene set, we first applied a Fisher's exact test to assess its overlap with sex-DE genes defined by specific thresholds (FD≥1.2 and *P*≤0.05, Fig. 1c, Supplementary Data 3; FD≥1.2 and *P*≤0.01 or *P*≤0.005, Supplementary Data 3). To corroborate these results and assess sexually dimorphic shifts in gene expression across the transcriptome without applying arbitrary thresholds, we also used a binomial test to assess each gene set's distribution into two possible outcomes: higher expression in males (FD>1, any *P* value), or higher expression in females (FD<1, any *P* value). We found no evidence from either test for significant sex-differential expression of any of these diverse sets of ASD risk genes in the adult cerebral cortex, providing no evidence supporting hypothesis 1 (Fig. 1d and Supplementary Data 3).

Next, we tested gene sets with evidence of ASD-associated expression patterns in adult human cortex^23^,^24^ (Supplementary Data 2), neural cell type marker gene sets^25^,^26^,^27^ (Supplementary Data 4) and genes marking cell types of the adaptive immune system^34^ (Supplementary Data 5) for sex-differential expression. We include immune cell markers in this analysis, since recent work has suggested complementary roles for T cells and microglia in mediating behaviour, specifically pain responses, in female versus male animals^38^. The ASD-associated gene sets include genes differentially expressed in post-mortem cortex from subjects diagnosed with ASD^24^, and two ASD-associated co-expression modules from this same study (asdM12_V_, downregulated in ASD and enriched for genes with neuronal and synaptic functions; and asdM16_V_, upregulated in ASD and enriched for genes involved in immune and inflammatory responses)^24^. We also tested three ASD-associated co-expression modules from a subsequent RNA-seq study by Gupta *et al.*^23^, which used a larger, but overlapping set of individuals (asdM1_G_, downregulated in ASD, and asdM6_G_, upregulated in ASD, both modules are enriched for neuronal markers and synaptic genes and overlap with the asdM12_V_ module^24^; asdM5_G_, which is upregulated in ASD, enriched for M2-state microglial genes, and overlaps with the asdM16_V_ module^24^). These DE genes and co-expression modules comprise large gene sets that were coherently altered in samples of ASD cases harbouring different genetic aetiologies. Therefore, these sets are likely to represent the downstream consequences of deleterious variants in risk genes, an upstream or ongoing background of molecular risk for ASD, or a secondary response to alterations in brain function that accompany ASD, any of which might be sexually dimorphic.

In contrast to the ASD risk genes, we observe significant enrichment and depletion for neural cell type markers and gene sets dysregulated in post-mortem brain from autistic subjects^24^. Among genes expressed at significantly higher levels (FD≥1.2, *P*<0.05) in males than in females (male-DE), we find a sevenfold enrichment of ASD-upregulated genes (7.0-fold, Fisher-adjusted *P* (*P*_Fisher_adj_)=1.5e-04; Fig. 1c and Supplementary Data 3). Among genes upregulated in ASD post-mortem cortex (ASD-up)^24^, 67% show higher expression in males than in females (male-higher, 21% shift from background expectation, binomial test-adjusted *P* (*P*_Binom_adj_)=6.9e-03, Fig. 1d). Genes belonging to the ASD-upregulated module asdM16_V_ also show enrichment for significantly male-DE genes (5.2-fold, *P*_Fisher_adj_=1.8e-10, Fig. 1c) and a significant shift towards male-biased expression overall (73% male-higher, 28% shift, *P*_Binom_adj_=4.9e-21, Fig. 1d). The related co-expression module asdM5_G_ from Gupta *et al.*^23^ also shows this male-biased shift in expression (57% male-higher, 11% shift, *P*_Binom_adj_=9.2e-08, Fig. 1d). It is critical to note that this apparent concordance between sex and ASD status is not the result of a male skew or lack of sex balance in the cases and controls used by either post-mortem ASD study^23^,^24^, as all samples consisted predominantly of males (Voineagu *et al.*^24^: 4/16 female ASD cases and 1/16 female controls; Gupta *et al.*^23^: 8/32 female ASD cases and 9/41 female controls). Both of these published studies use multiple approaches to show that sex confounds do not drive the observed ASD-associated gene expression changes in brain^23^,^24^.

The ASD-upregulated gene list asdM16_V_, and asdM5_G_ are associated with astrocyte and microglial function; asdM16_V_ overlaps significantly with human microglial and astrocyte co-expression modules identified in neurotypical cerebral cortex^24^,^39^ and asdM5_G_ is enriched for gene expression signatures from M2 microglia^23^. Consistent with this, we also observe significant enrichments among male-DE genes for two independently generated sets of microglia markers (Albright *et al.*^25^: 2.1-fold, *P*_Fisher_adj_=0.029; Zeisel *et al.*^27^: 6.4-fold, *P*_Fisher_adj_=1.8e-12, Fig. 1e, Supplementary Data 3) and for two independently generated sets of astrocyte markers (Cahoy *et al.*^26^: 2.4-fold, *P*_Fisher_adj_=1.4e-08; Zeisel *et al.*^27^: 4.2-fold, *P*_Fisher_adj_=1.0e-04, Fig. 1e); this is corroborated by significant shifts towards male-biased expression for both astrocyte gene sets and the microglial markers generated by single-cell RNA-seq^27^ (Cahoy *et al.*^26^, astrocytes: 60% male-higher, 15% shift, *P*_Binom_adj_=8.5e-37; Zeisel *et al.*^27^, astrocytes: 69% male-higher, 23% shift, *P*_Binom_adj_=4.2e-09; microglia: 64% male-higher, 19% shift, *P*_Binom_adj_=1.7e-07, Fig. 1f).

In contrast, the forebrain neuronal marker gene set^26^ is significantly enriched for genes expressed at significantly higher levels in females than in males (FD≥1.2, *P*<0.05; female-DE; 1.8-fold, *P*_Fisher_adj_=0.019, Fig. 1e) and shifted towards female-biased expression overall (64% with higher expression in females (female-higher), 9% shift, *P*_Binom_adj_=3.3e-11, Fig. 1f). Similarly, genes in the ASD-downregulated, neuronal and synaptic module asdM12_V_ show enrichment for female-DE genes (2.7-fold, *P*_Fisher_adj_=5.8e-03, Fig. 1c), while the ASD-downregulated module asdM1_G_ shows depletion of male-DE genes (0.37-fold, *P*_Fisher_adj_=8.1e-04, Fig. 1c); both show a significant shift towards female-biased expression overall (asdM12_V_: 70% female-higher, 16% shift, *P*_Binom_adj_=1.0e-07; asdM1_G_: 64% female-higher, 10% shift, *P*_Binom_adj_=8.2e-14, Fig. 1d). No T- or B-cell modules showed any significant enrichment with sex-DE genes or any significant shift towards male- or female-biased expression (Supplementary Fig. 1a).

### Comparison of sex- and ASD-differential expression patterns

**Figure 2:**
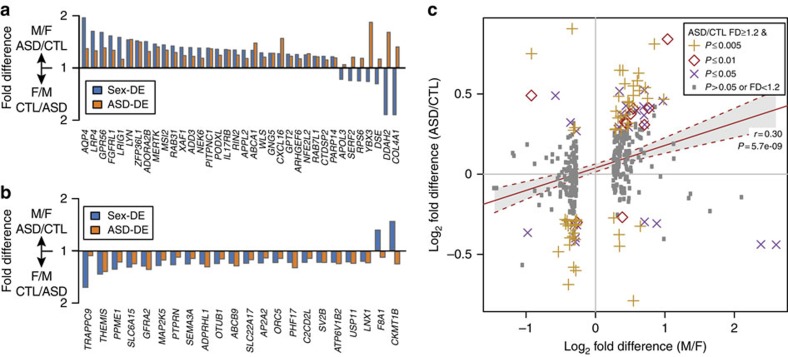
Sex-differential expression parallels gene expression patterns in ASD brain. Bar plots show FDs by sex and ASD status of sex-DE genes (FD≥1.2 and *P*<0.05) that also belong to ASD-associated co-expression modules^24^. (**a**) Module asdM16_V_, which is significantly upregulated in ASD and enriched for genes involved in the inflammatory response and immune system functions, and (**b**) module asdM12_V_, which is significantly downregulated in ASD and enriched for genes with neuronal and synaptic functions. (**c**) FDs of sex-DE genes (FD≥1.2, *P*<0.05, 374 genes) by sex and status. Best-fit line and its 95% confidence interval are shown. CTL, control from ASD expression study; F, female; M, male.

We were particularly interested in the sex-DE enrichment in the ASD-associated co-expression modules from post-mortem brain, asdM16_V_ and asdM12_V_ (ref. 24). Previous studies have demonstrated that co-expression modules frequently correspond to neural cell types and coherent biological functions^39^,^40^; thus, sex-DE enrichments in these modules may most directly implicate pathways involved in sex-biased ASD risk. Strikingly, we observe that all 36 sex-DE genes that are also members of asdM16_V_ are expressed at higher levels in ASD than in control cortex^24^, 29 of which are also expressed at higher levels in neurotypical male than in female cortex (Fig. 2a). We also observe that all of the 22 sex-DE genes in asdM12_V_ are expressed at lower levels in ASD than in control cortex, and 20 of these are also expressed at higher levels in females compared with males (Fig. 2b). In short, we find concordant directionality of differential expression by sex and ASD status, with asdM16_V_ sex-DE genes expressed at higher levels in ASD and typical males and asdM12_V_ sex-DE genes expressed at higher levels in controls and typical females.

To assess the extent of these parallel expression patterns across the transcriptome, we compared the FDs from sex-DE genes with FDs observed in the differential expression analysis of ASD cortex^24^. For the 374 sex-DE genes (FD≥1.2, *P*≤0.05) that were also tested in the ASD-DE analysis, we observe a highly significant positive correlation between the sex-differential and ASD-differential FDs (*r*=0.30, *P*=5.7e-09, Fig. 2c), as well as for the subset of genes differentially expressed at FD≥1.2 and *P*≤0.05 in both comparisons (*r*=0.29, *P*=5.7e-04, *N*=90 genes). We note that among the 47 genes expressed at significantly higher levels in both males and in ASD, 22 are asdM16_V_ members^24^ and 19 are astrocyte markers^26^ (16 belong to both sets). Among the 25 genes expressed at significantly higher levels in both females and in unaffected controls, 11 are asdM12_V_ members^41^ and 12 are neuron markers^14^ (5 belong to both sets). These results suggest that the parallels in sex-differential and ASD-differential expression direction extend beyond asdM16_V_ and asdM12_V_, and that this pattern may be more broadly indicative of sexually dimorphic transcriptomes originating from specific neural cell types.

### Independent replication of adult sex-differential expression

**Figure 3:**
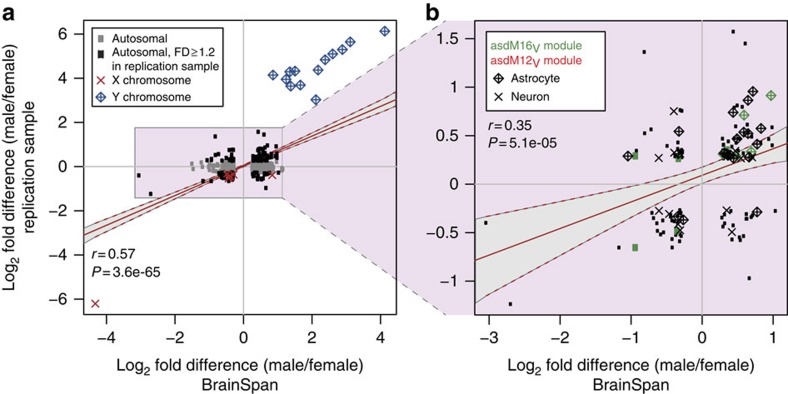
Male-biased expression of astrocyte and asdM16_V_ module genes in an independent sample. (**a**) FD for all sex-DE genes from the BrainSpan sample also tested in the replication set (BrainSpan FD≥1.2, *P*<0.05, 733 genes; grey points mark genes with FD<1.2 in the replication sample), and for (**b**) autosomal sex-DE genes from BrainSpan with FD≥1.2 in the replication sample (129 genes). Best-fit lines and 95% confidence intervals are shown on plots.

To validate these observations, we next analysed RNA-seq data from an independent sample of adult cortex tissue (16–56 years, Supplementary Table 1) for sex-differential expression patterns. Using this smaller data set, we identify 65 genes at *P*<0.005, 98 genes at *P*<0.01 and 519 genes at *P*<0.05 (Supplementary Fig. 2a,b and Supplementary Data 1). Overall, sex-DE genes from the BrainSpan data were expressed at comparable levels and with positively correlated fold differences in this independent sample (expression levels: *r*=0.78, *P*=2.9e-150; log_2_(FD)s, *r*=0.57, *P*=3.6e-65; Fig. 3a). We observed no over-representations of ASD risk genes among significantly sex-DE genes (FD≥1.2, *P*<0.05) from these data (Supplementary Fig. 2d and Supplementary Data 6); however, FMRP targets were more likely to show higher expression in females than in males (62% female-higher, 13% shift, *P*_Binom_adj_=2.9e-10, Supplementary Fig. 2d). Oligodendrocyte markers and endothelial cell markers also showed shifts towards female-biased expression (Cahoy *et al.*^26^ oligodendrocytes: 56% female-higher, 9% shift, *P*_Binom_adj_=1.5e-11; Zeisel *et al.*^27^ oligodendrocytes: 59% female-higher, 15% shift, *P*_Binom_adj_=5.8e-07; endothelial cells^27^: 55% female-higher, 11% shift, *P*_Binom_adj_=5.7e-03, Supplementary Fig. 2f). We also found depletion of male-DE genes in asdM1_G_ (0.26-fold, *P*_Fisher_adj_=0.027, Supplementary Fig. 2c), but enrichments for asdM16_V_ genes (4.7-fold, *P*_Fisher_adj_=2.8e-03; 64% male-higher, 11% shift, *P*_Binom_adj_=5.2e-03, Supplementary Fig. 2c,d), asdM6_G_ genes (58% male-higher, 7% shift, *P*_Binom_adj_=0.014, Supplementary Fig. 2d), astrocyte marker genes (Cahoy *et al.*^26^: 61% male-higher, 10% shift, *P*_Binom_adj_=1.7e-16; Zeisel *et al.*^27^: 69% male-higher, 18% shift, *P*_Binom_adj_=6.6e-06, Supplementary Fig. 2f) and ependymal cell markers (60% male-higher, 9% shift, *P*_Binom_adj_=0.027, Supplementary Fig. 2f). Again, we find no sex-differential expression signal in T- and B-cell modules (Supplementary Fig. 1b and Supplementary Data 6). Of the 164 genes expressed more highly in males with a FD≥1.2 in both adult data sets, 24 are asdM16_V_ members (8.8-fold enrichment, *P*=5.5e-14) and 70 are astrocyte markers (10.3-fold enrichment, *P*=3.3e-35; 14 of these genes are belong to both sets), further supporting the characterization of astrocytic genes and ASD-upregulated, microglial-function-enriched asdM16_V_ genes, as displaying male-biased expression in the adult human cortex (Fig. 3b).

### Sex-differential expression in prenatal human cortex

One possible explanation for the lack of enrichment of ASD risk genes among sex-DE genes from adult brain is that ASD risk genes may be acting primarily during fetal brain development^30^,^31^. To address this, we next analysed array expression data from 86 neocortex samples from 8 subjects (4 females) between 16–22 post-conception weeks^29^ (Supplementary Table 1). This prenatal epoch follows the mid-gestation peak in testosterone secretion from males' differentiated testes^42^,^43^ and thus captures a stage during which steroid hormones are likely to be exerting organizational effects.

**Figure 4:**
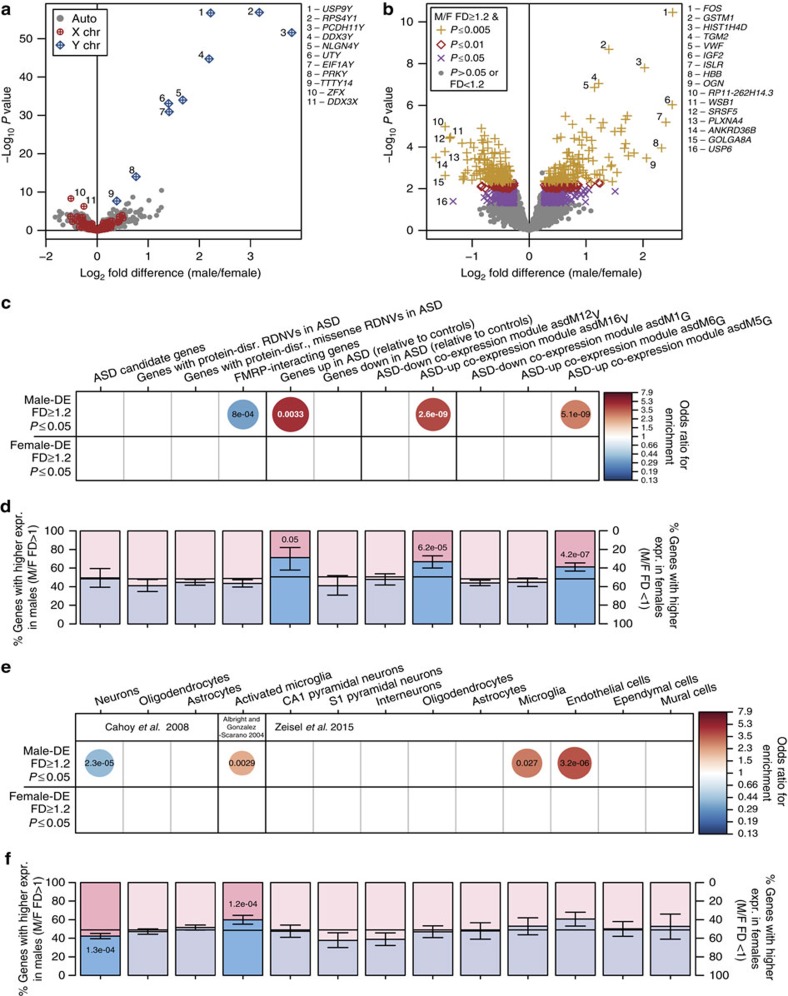
Microglia and astrocyte markers and genes upregulated in ASD brain tend toward higher expression in prenatal male brain. (**a**) Volcano plot for all 9,889 transcripts expressed in the prenatal sample (*n* male=43 samples from 4 subjects, *n* female=43 samples from 4 subjects). (**b**) Subset of the plot in **a** for the 9,532 autosomal transcripts. (**c**) Enrichment for ASD risk genes and ASD-associated gene expression patterns, and (**e**) neural cell type markers, within male-DE (509 genes) and female-DE (528 genes) gene sets by Fisher's exact test; circle size and colour indicate the odds ratio of all significant overlaps (Bonferroni-adjusted *P* value <0.05); overlaid text displays the adjusted *P* value for each enrichment. (**d**) Shifts in the distribution of sex-differential expression direction for genes in each ASD risk or ASD expression sets, and (**f**) neural cell type markers. Blue and pink bars display the proportions of each gene set that have higher expression in males (FD>1) or females (FD<1); whiskers note 95% confidence intervals; horizontal black lines note the proportion of male- and female-higher genes in the corresponding background gene set. Overlaid text displays significant Bonferroni-adjusted *P* values from the binomial test. chr, chromosome; disr., disrupting; expr., expression; F, female; M, male.

We find 306 sex-DE genes at *P*<0.005, 440 sex-DE genes at *P*<0.01 and 1,037 sex-DE genes at *P*<0.05, with 96 genes sex-DE at an adjusted *P*<0.05 (Fig. 4a,b and Supplementary Data 1). Results from over-representation analysis for the gene sets of interest within these prenatally sex-DE genes mirror those in the adult data: there are no significant enrichments for ASD risk gene sets, and FMRP interactors are significantly depleted within male-DE genes (0.33-fold, *P*_Fisher_adj_=8.0e-04, Fig. 4c, Supplementary Data 7).

As in adult brain, genes expressed more highly in prenatal male cortex show robust enrichments for post-mortem cortex ASD-upregulated genes (4.6-fold, *P*_Fisher_adj_=3.3e-03, Fig. 4c), asdM16_V_ genes (4.0-fold, *P*_Fisher_adj_=2.6e-09; 67% male-higher, 16% shift, *P*_Binom_adj_=6.2e-05, Fig. 4c), asdM5_G_ genes (2.9-fold, *P*_Fisher_adj_=5.1e-09; 61% male-higher, 13% shift, *P*_Binom_adj_=4.2e-07, Fig. 4c,d), microglia markers (Albright *et al.*^25^: 2.1-fold, *P*_Fisher_adj_=2.8e-03; 60% male-higher, 11.4% shift, *P*_Binom_adj_=1.2e-04; Zeisel *et al.*^27^: 2.9-fold, *P*_Fisher_adj_=0.027, Fig. 4e,f), endothelial markers (3.7-fold, *P*_Fisher_adj_=3.2e-06, Fig. 4e) and a significant depletion of forebrain neuron marker genes (0.41-fold, *P*_Fisher_adj_=2.3e-05; 58% female-higher, 7% shift, *P*_Binom_adj_=1.3e-04, Fig. 4e,f). As in adult, T- and B-cell modules show no enrichments or depletions of sex-DE genes, nor any sex-skewed expression shifts (Supplementary Fig. 1c and Supplementary Data 7).

Taken together, these findings fail to support the hypothesis that ASD risk genes are differentially expressed by sex in the human cerebral cortex. Rather, we observe that sex-DE genes are enriched among specific classes of changes observed in post-mortem cortex from ASD patients, which is indicative of shared biology between ASD and typical sexual dimorphisms. These patterns of sex-DE enrichment follow what is observed in ASD cortex, with higher expression of microglial and astrocyte genes (exemplified by asdM16_V_ and asdM5_G_) in the more susceptible male population, and higher expression of a cohort of synaptic genes (exemplified by asdM12_V_), in the more protected, female population.

## Discussion

Autism risk is sexually dimorphic, and genetic studies are most consistent with a female protective effect. To begin to explore potential mechanisms mediating sex-differential genetic risk, we tested the hypothesis that ASD risk genes would show sexually dimorphic expression in the cerebral cortex, a region that plays a major role in complex human cognitive phenotypes, including social behaviour, and where many ASD genes are expressed^30^,^31^. We observe both in adult and fetal brain that while some ASD risk genes do show sex-differential expression, overall this is not beyond what would be expected by chance. Furthermore, these sex-neutral expression patterns are evident in the mid-fetal cortex, a spatiotemporal point of convergence for ASD risk genes' expression^30^,^31^.

Instead, we find support for a second hypothesis: that genes whose expression patterns are altered in post-mortem autistic brain, which highlight downstream or interacting pathways, but not ASD risk genes themselves, are differentially expressed by sex. The convergence of male-DE genes with these downstream pathways, but not with risk genes or neuronal markers, is consistent with the evidence presented by Voineagu *et al.*^24^: asdM16_V_ is upregulated in ASD brain and enriched for immune system function and inflammatory response genes, but not for common ASD susceptibility variants. The asdM16_V_ module is therefore interpreted as representing a secondary, likely downstream effect of genetically causal perturbations. Whether a secondary effect of genetic variants or a result of independent mechanisms, it may be that expression of this gene set beyond a certain threshold is detrimental to neural development and function. Our findings show that typical males land closer to this putative threshold than females do, potentially implicating these genes expressed at higher levels in the male and ASD cortex in mechanisms driving male-biased ASD risk. Furthermore, to the extent that asdM16_V_ upregulation contributes to ASD pathophysiology, this module may serve as a useful target for pharmacological treatments that could modulate the effects of heterogeneous risk variants acting upstream.

Alternatively or concordantly, it may also be the case that ASD-protective genes are expressed at a higher level in females. Genes in the neuronal- and synapse function-enriched asdM12_V_ module may represent such a protective gene set, as these genes are downregulated in ASD post-mortem cortex^24^. Genes belonging to asdM12_V_ do show higher expression in adult female cortex from the BrainSpan sample (Fig. 1c,d), though neither the replication adult sample nor the prenatal sample show this same female skew. Alternatively, the relatively sex-neutral expression of asdM12_V_ genes in contrast to asdM16_V_ may simply reflect the absence of a particular ASD pathophysiological process in neurotypical samples.

We also observed a correlation in the relative FDs of sex-DE genes and ASD-DE genes that extends beyond the asdM16_V_ module, such that genes expressed more highly in typical male cortex also tend to be more highly expressed in ASD cortex, while genes more highly expressed in typical female cortex also tend to be expressed at lower levels in ASD cortex^24^. Again, we emphasize this agreement in fold change direction between sex and ASD status is not likely the result of a male-confounded analysis by Voineagu *et al.*^24^ or Gupta *et al.*^23^, as cases and controls were sex balanced, and any potential sex effects were also regressed out in those studies. Interestingly, the gene expression patterns we observe fit squarely with hypotheses derived from both the extreme male brain theory and from the concept of female protective factors in ASD: male-DE (ASD-upregulated) genes could act to increase risk, while female-DE (ASD-downregulated) genes could have protective functions. However, we caution that FDs do not indicate the underlying mechanism of gene regulation (that is, active upregulation in males can return the same result as repression in females). We cannot yet conclude that parallel expression differences between the sexes and in ASD are derived from the same regulatory mechanisms, but this work provides a starting point for their study. Additional experiments that include sufficient numbers of cases and controls of both sexes will be necessary to tease apart how sex and ASD status interact to affect gene expression, for example, to determine whether male-DE genes are expressed at even higher levels in ASD cases.

Astrocyte and microglial markers are also reliably enriched among male-DE genes in both adult and prenatal cortex. We recognize two possible explanations for this: (1) a greater proportion of astrocytes and microglia to neurons in male cortex results in higher measured gene expression in males; or (2) males' cortical astrocytes and microglia are more transcriptionally active than females'. Aside from early, tenuous observations of greater cortical neuron density in males^44^, sex differences in the cellular composition of human cortex have not been sufficiently characterized to determine if there is a greater number of certain glial subtypes in male cortex. In contrast, there is clear evidence for sexually dimorphic astrocyte morphology in animals, particularly in hypothalamic nuclei, with male astrocytes showing a greater number of longer and more branched processes^45^,^46^,^47^. In addition, sex steroid hormones may play a role in astrocyte differentiation and regulation, as oestrogen receptors are expressed in hypothalamic and hippocampal astrocytes^48^,^49^, and female animals exposed to testosterone neonatally show male-typical astrocyte morphology^46^. Given the participation of astrocytes in the modulation of neurotransmission^50^,^51^ and of microglia and astrocytes in synapse formation and function^52^,^53^,^54^,^55^, it is plausible that sexual dimorphisms in astrocyte and/or microglia number or function would lead to sex-differential effects on neuronal connectivity^56^. It is also interesting to consider that numerous ASD risk genes function at the synapse^7^,^9^,^57^,^58^,^59^, and that as a third synaptic component along with pre- and postsynaptic neurons, sexually dimorphic astrocytes and/or microglia are well positioned to broadly influence the effects of upstream, heterogeneous risk variants.

We also acknowledge several limitations of our study. First, our analyses utilize gene expression data from the human cerebral cortex, a region that other studies have indicated is a convergent area where many ASD risk genes likely exert their action^30^,^31^. However, other brain regions not sampled in our data sets, such as the hypothalamic nuclei, are known to be robustly sexually dimorphic^60^. It may be that ASD risk genes are differentially expressed by sex in these sexually dimorphic regions, or that it is the connections between these regions and other parts of the brain where the sex-differential risk-modulatory mechanisms act (as opposed to purely within-cortex interaction at the level of molecular pathways and local cell–cell connections). Assessment of additional brain regions, including hypothalamic nuclei and other subcortical structures, in sufficiently powered test and replication sets will be useful for addressing this important question, once such data are available.

The mechanism(s) of the sex-biased expression observed here are not known, but present significant opportunities for understanding ASD's sex-biased pathogenesis. For example, transcription of sex-DE genes identified here may be regulated by sex-differential factors, such as transcription factors from the sex chromosomes, or by sex steroid receptor binding to hormone response elements upstream from transcription start sites. Chromatin immunoprecipitation experiments in human neural tissue coupled with functional assays in model systems will be needed to thoroughly characterize binding sites for these regulatory elements throughout the genome.

Alternatively, the sex differences we have observed may not result from differential transcription regulation, but instead from sex differences in the proportions of different neural cell types within male and female tissue. The overlap between male-DE genes and astrocyte and microglial marker genes is consistent with this possibility, although the processes that lead to sex differences in cell type proliferation or differentiation are unclear. Quantitative histological studies of male and female human brain will be critical to characterize the extent to which cell type composition differs between the sexes, and neurodevelopmental follow-up will be required to demonstrate how these differences arise.

The extent to which the convergent transcriptional changes previously observed in ASD cortex^23^,^24^ represent causal aetiologies versus consequences of brain dysfunction also remains to be delineated^61^. In the case of ASD-associated co-expression modules enriched for risk variants, such as asdM12_V_, enrichment of genes carrying ASD risk variants provides evidence for a causal, primary role in ASD aetiology. However, ASD-associated co-expression modules that lack enrichment for genetic risk variants, such as asdM16_V_, may be linked to ASD in one of several ways: (1) They reflect direct downstream consequences of variants in, or dysregulation of, primary, ‘causal' genes (for example, asdM12_V_) that actively participate in the pathophysiological processes that shape and maintain an autistic-functioning brain; (2) they represent risk pathways that are independent of primary risk genes, for example, consequences of an environmental exposure, that also actively participate in ASD's aetiology; or (3) they exemplify consequences of living with ASD to the brain, which may modify the presentation of ASD or its comorbidities, but that do not have a role in ASD's aetiology. For example, the gene expression signature captured by non-variant-enriched, co-expression module asdM16_V_, which is upregulated in post-mortem adult autistic cortex, may reflect the impact of chronic social stress experienced across the lifespan. If male and female brains respond differently to stress^62^, then the concordance we observe between these gene expression signatures may be tangential to ASD's primary pathophysiology. Though, one would expect that such stress would be cumulative over the lifespan, so that a stress-driven gene expression profile would increase with experience and age. Neither of the ASD-associated co-expression modules is significantly correlated with age^23^,^24^, making this explanation less likely. Nevertheless, extensive characterization of animal models of ASD genetic risk variants, including gene expression patterns, may help to determine whether expression signatures like these are purely consequential, or if they are evident during early development and play a role in ASD aetiology.

In any case, the sex-DE patterns that we observe in multiple, independent samples demonstrate that currently identified ASD risk genes are not innately sex-differentially expressed in the developing or adult human cerebral cortex. Instead, it is the molecular, cellular or circuit-level context in which ASD risk genes operate that is likely responsible for modulating ASD liability in a sex-differential manner. This notion fits with the remarkable consistency of the male bias in ASD prevalence, despite ongoing evidence of considerable genetic heterogeneity in ASD. Study of astrocyte–neuronal and microglia–neuronal synapse interactions, and study of the causes and functional consequences of higher expression of astrocyte and microglial genes in male brain and synaptic genes in female brain, are needed to determine how sex-differential functioning of these pathways impact sexually dimorphic risk for a variety of neurodevelopmental disorders, including ASD.

## Methods

### Adult BrainSpan data set

Developmental RNA-seq data from the BrainSpan project^28^ summarized to Gencode 10 (ref. 41) gene-level reads per kilobase million mapped reads (RPKM) were used. Data were normalized for GC content, using conditional quantile normalization^63^, batch-corrected for processing site using ComBat^64^ and log_2_-transformed (log_2_(RPKM+1)). Samples from regions of the frontal, temporal and parietal cortex from subjects aged 13–40 years, with RNA integrity number (RIN) of at least 8.0, were retained for this analysis. Non-expressed genes defined as having a log_2_-transformed RPKM expression level of <1 in more than 50% of all male or female samples were removed. Outlier samples were identified by evaluating inter-sample correlations and hierarchical clustering, first within each sex, and then on the full data set (samples of both sexes considered together); samples with inter-sample correlations >2.5 s.d.'s from the mean were removed. The data were then quantile normalized to mitigate the effects of systematic differences in the distribution of expression levels across samples on the differential expression analysis results. After gene filtering and outlier removal, 72 samples (29 from males and 43 from females) and 16,843 expressed genes remained. To balance the male and female sample sets, we then matched a subset of the female samples to the male samples by age and brain region, and again filtered for expressed genes. The final data set consisted of 58 samples from 10 subjects (29 samples from 5 subjects of each sex; Supplementary Table 1) and 16,392 genes (Supplementary Data 1).

### Adult replication data set

The adult replication sample comprised seven frontal and temporal cortex samples from five male individuals matched to seven cortical samples from five female individuals with no history of neuropsychiatric or neurological diagnoses. Male and female samples were matched for age, post-mortem interval (PMI) and brain region. These samples were a subset of a larger set acquired from the Harvard Brain and Tissue Bank and the Eunice Kennedy Shriver National Institute for Child Health and Human Development Brain and Tissue Bank for Developmental Disorders following the tissue acquisition policies of the respective brain banks.

Approximately 100 mg of tissue from each sample was dissected on dry ice; RNA isolations were performed using the miRNeasy kit (Qiagen), RNA quality was quantified using the RIN^65^ and ribosomal RNA was depleted using the Ribo-Zero Gold kit (Epicentre). Remaining RNA was then size selected with AMPure XP beads (Beckman Coulter) and re-suspended, and sequencing libraries with indexed adaptors were prepared according to the Illumina TruSeq protocol. Fifty base-pair, paired-end reads were generated on a HiSeq 2000/2500, with 24 samples pooled per lane.

Reads were mapped to the human reference genome (hg19) using Gencode v18 annotations with TopHat2, allowing for up to 10 multiple mappings per read^41^,^66^. Output BAM files were filtered to ensure that every read had a valid pair, resulting in only paired-end reads (fragments) being used for downstream analyses. Transcript levels were quantified using Gencode v18 gene models at the union gene model level using HT-seq Counts^67^. We normalized these data for GC content biases using the cqn package in R (ref. 63), which resulted in log_2_(normalized FPKM) values, and ensured that there were no sample outliers with a summed sample correlation *Z*-score>2 (ref. 68). Genes not expressed in the subset of neurotypical samples selected for these analyses were identified (log_2_-transformed FPKM expression level <1 in more than 50% of all male or female samples) and removed. Outlier samples with inter-sample correlations >2.5 s.d.'s from the mean, either within each sex or within the complete sample set, were removed. The expression data for the remaining samples were then quantile normalized. The final filtered data set consisted of 13 samples from 10 subjects (Supplementary Table 1), and 19,354 genes (Supplementary Data 1).

### Prenatal data set

Exon array data analysed by Kang *et al.*^29^ and downloaded from the Gene Expression Omnibus (GSE25219) were used for the assessment of prenatal gene expression. Only samples from subjects between 16 and 22 post-conception weeks from the frontal, temporal and parietal cortex, and with RIN of 8.0 or greater were used in this stage of analysis.

Probe set IDs from the downloaded data were matched to Ensembl Gene IDs from Gencode 10 using the biomaRt function in R. Non-expressed genes were defined as those genes with a log_2_-transformed median probe set intensity of <6 in >80% of male or female samples within the selected sample set, and were removed. Outlier samples were detected by evaluating inter-sample correlations and hierarchical clustering within each sex and within the complete data set, and samples with inter-sample correlation >2.5 s.d.'s from the mean were removed. Expression data for the remaining samples were quantile normalized. After these processing steps, 142 samples (43 from males and 99 from females) and 9,985 expressed genes remained. To balance the number of male and female samples for analysis, we then matched a subset of the female samples to the male samples on age and brain region, and again filtered for expressed genes. The final data set consisted of 86 samples from 8 subjects (43 samples from 4 subjects of each sex; Supplementary Table 1), and 9,889 genes (Supplementary Data 1).

### Differential expression analysis

Differential expression analyses for all data sets were performed using a linear mixed model and Bayesian *t*-tests as implemented in LIMMA^69^, a robust method for analysing small samples. For all analyses, the main contrast in the regression model was sex, and subject was included as a random effect to account for the non-independence of samples from the same individual brain (that is, different brain regions). Covariates were included, where available, in the model as fixed effects: RIN, age, PMI, cortical lobe and pH for the adult BrainSpan data; RIN, age, PMI and cortical lobe for the replication data; and RIN, age, PMI, cortical lobe and pH for the prenatal data. Per-subject average values were substituted for missing PMI and pH information. Genes with a FD magnitude of 1.2 or greater and an unadjusted *P* value of 0.005, 0.01 or 0.05 were called as differentially expressed by sex (sex-DE; Supplementary Data 1).

### Annotation gene sets

ASD candidate genes were selected from the SFARI Gene Autism Database^32^ and filtered to those genes classified as syndromic (category S) or with evidence levels between 1–4 for a set of 153 genes. To also investigate a risk gene set based on evidence from an unbiased, experimental screen, we used the set of genes with rare *de novo* variants (RDNVs) in sporadic ASD cases from exome sequencing of families from the Simons Simplex Collection^8^. All genes with RDNVs in autistic probands were compiled and classified as protein-disrupting (nonsense, splice site or frameshift mutations), missense or silent variants. Here we use the gene set with protein-disrupting variants (353 genes) and the expanded gene set with either protein-disrupting or missense variants (1,771 genes). We also evaluated the set of transcripts that are binding targets of FMRP, a functionally defined gene set that is enriched for ASD risk genes^70^. Here we tested the 725 human orthologues of FMRP target genes as characterized in mouse brain^33^ (Supplementary Data 2).

Seven gene sets with ASD-associated expression patterns in adult human cortex were also tested. From a 2011 study by Voineagu *et al.*^24^, we tested genes expressed at significantly (1) higher (FD≥1.2, *q*-value<0.1, 88 genes) or (2) lower levels in ASD than in control cortex (125 genes); (3) ASD-associated, unsigned co-expression modules from weighted gene co-expression network analysis, asdM12_V_ (downregulated in ASD, 414 genes); and (4) asdM16_V_ (upregulated in ASD, 361 genes). From a 2014 study by Gupta and colleagues^23^ that used a larger, but overlapping, sample of individuals as Voineagu *et al.*^24^, but which analysed BA19, a different cortical region, we tested several ASD-associated weighted gene co-expression network analysis modules, including (5) asdM1_G_ (downregulated in ASD, 1,643 genes), (6) asdM6_G_ (upregulated in ASD, 667 genes) and (7) asdM5_G_ (upregulated in ASD, 759 genes) (Supplementary Data 2).

Since gene expression patterns in the brain often reflect the transcriptional activity of distinct neural cell types^39^,^40^, we also culled gene sets marking different cell types from several studies in the literature. From a microarray study of sorted cell types in mouse forebrain^26^, we tested markers for neurons (1,489 genes), oligodendrocytes (1,626 genes) and astrocytes (1,968 genes). From a microarray study of human microglial cells in culture^25^, we tested genes with at least a twofold upregulation in activated, microglia-enriched, mixed glial cultures (∼60% microglia) (773 genes). From a single-cell RNA-seq study of mouse somatosensory cortex and hippocampus^27^, we tested markers for CA1 pyramidal neurons (355 genes), S1 pyramidal neurons (235 genes), interneurons (319 genes), oligodendrocytes (397 genes), astrocytes (216 genes), microglia (368 genes), endothelial cells (312 genes), ependymal cells (402 genes) and mural cells (pericytes and vascular smooth muscle cells, 137 genes) (Supplementary Data 4).

Recent evidence has also suggested complementary roles for microglia and immune cells in mediating behaviour in male versus female animals^38^. To evaluate whether adaptive immune cells contribute to the sex-differential gene expression that we observe in the brain, we tested co-expression modules generated by the Immunological Genome Project from mouse haematopoietic cells that are associated with T- and B-cell lineage regulator genes^34^: T-cell module 18 (106 genes); gamma delta T-cell module 65 (30 genes); gamma delta, natural killer T and T4 module 56 (58 genes); alpha beta T-cell module 57 (52 genes); B-cell module 33 (125 genes); B-cell module 61 (38 genes); and B-cell module 76 (15 genes; Supplementary Data 5).

For the enrichment analyses, we used the following gene sets as background: for SFARI candidate genes, genes with RDNVs in ASD cases and activated microglial markers, we used expressed genes with a Gencode biotype annotation of ‘protein coding'; for gene sets with ASD-associated expression patterns and for immune cell markers, we used expressed genes also tested in the corresponding source publication^23^,^24^,^34^; and for FMRP target genes^33^ and cell type markers from Cahoy *et al.*^26^ and Zeisel *et al.*^27^, we used the genome-wide set of one-to-one human–mouse orthologues as background (Supplementary Data 8).

For all background and gene sets of interest, gene identifiers were converted to Ensembl Gene IDs in Gencode using the biomaRt package in R to allow for unambiguous comparisons between genes from different data sources. Gene set membership, background set membership and sex-DE categorization for all expressed genes tested for differential expression in the three data sets are recorded in Supplementary Data 9.

### Over-representation analysis

To evaluate the enrichment of gene sets of interest among sex-DE genes, we applied a two-sided Fisher's exact test separately to sex-DE gene sets expressed more highly in males (male-DE) and in females (female-DE) at three *P* value thresholds (unadjusted *P*<0.005, 0.01 and 0.05, Supplementary Data 3, 6 and 7) in R. *P* values from the Fisher's exact tests run within each data set were adjusted for 62 tests performed against the 31 gene sets of interest (4 ASD risk gene sets, 7 ASD expression gene sets, 13 neural cell type marker sets and 7 adaptive immune cell marker sets) at each of the three significance levels for the sex-DE input genes.

To further investigate patterns of sexually dimorphic expression within each gene set of interest without applying arbitrary thresholds to define sex-DE genes, we also assessed the distribution of the genes in each set into two mutually exclusive categories: higher expression in males (positive log_2_(fold difference), at any *P* value); or higher expression in females (negative log_2_(fold difference), at any *P* value). We then applied a two-sided binomial test against the distribution of male-higher and female-higher genes in the corresponding background gene set (Supplementary Data 3, 6 and 7). *P* values from all binomial tests within each data set were Bonferroni-corrected for the 31 gene sets tested.

## Additional information

**Accession codes:** Gene expression data analysed in this study come from publicly available resources (adult BrainSpan RNA-seq data, www.brainspan.org; prenatal array data, GEO accession GSE25219; and adult replication RNA-seq data, GEO accession GSE76852). Computer code used in this study is available on request.

**How to cite this article:** Werling, D. M. *et al.* Gene expression in human brain implicates sexually dimorphic pathways in autism spectrum disorders. *Nat. Commun.* 7:10717 doi: 10.1038/ncomms10717 (2016).

## Supplementary Material

Supplementary Information Supplementary Figures 1-2 and Supplementary Table 1

Supplementary Data 1 Sex-differential expression results from all three data sets: adult BrainSpan RNA-seq, adult replication set RNA-seq, prenatal exon array.

Supplementary Data 2 Tables for all ASD-risk and ASD-associated gene sets tested for enrichment of sex-differential expression.

Supplementary Data 3 Over-representation analysis results for adult BrainSpan RNA-seq data set.

Supplementary Data 4 Tables for all neural cell type marker gene sets tested for enrichment of sex-differential expression.

Supplementary Data 5 Tables for all adaptive immune system cell type marker gene sets tested for enrichment of sex-differential expression.

Supplementary Data 6 Over-representation analysis results for adult replication RNA-seq data set.

Supplementary Data 7 Over-representation analysis results for prenatal exon array data set.

Supplementary Data 8 Tables for all gene sets used as background in over-representation analyses.

Supplementary Data 9 Membership in gene sets of interest, background gene sets, and sex-differentially expressed gene sets for all expressed genes from each of the three data sets analyzed.
