# Supplementary material for: Gene expression in human brain implicates sexually dimorphic pathways in autism spectrum disorders
Source: Nat Commun. 2016 Feb 19;7:10717. doi: 10.1038/ncomms10717 (PMC4762891; doi:10.1038/ncomms10717)
Supplement: Supplementary Information — Supplementary Figures 1-2 and Supplementary Table 1 [file ncomms10717-s1.pdf]

Supplementary Figure-1

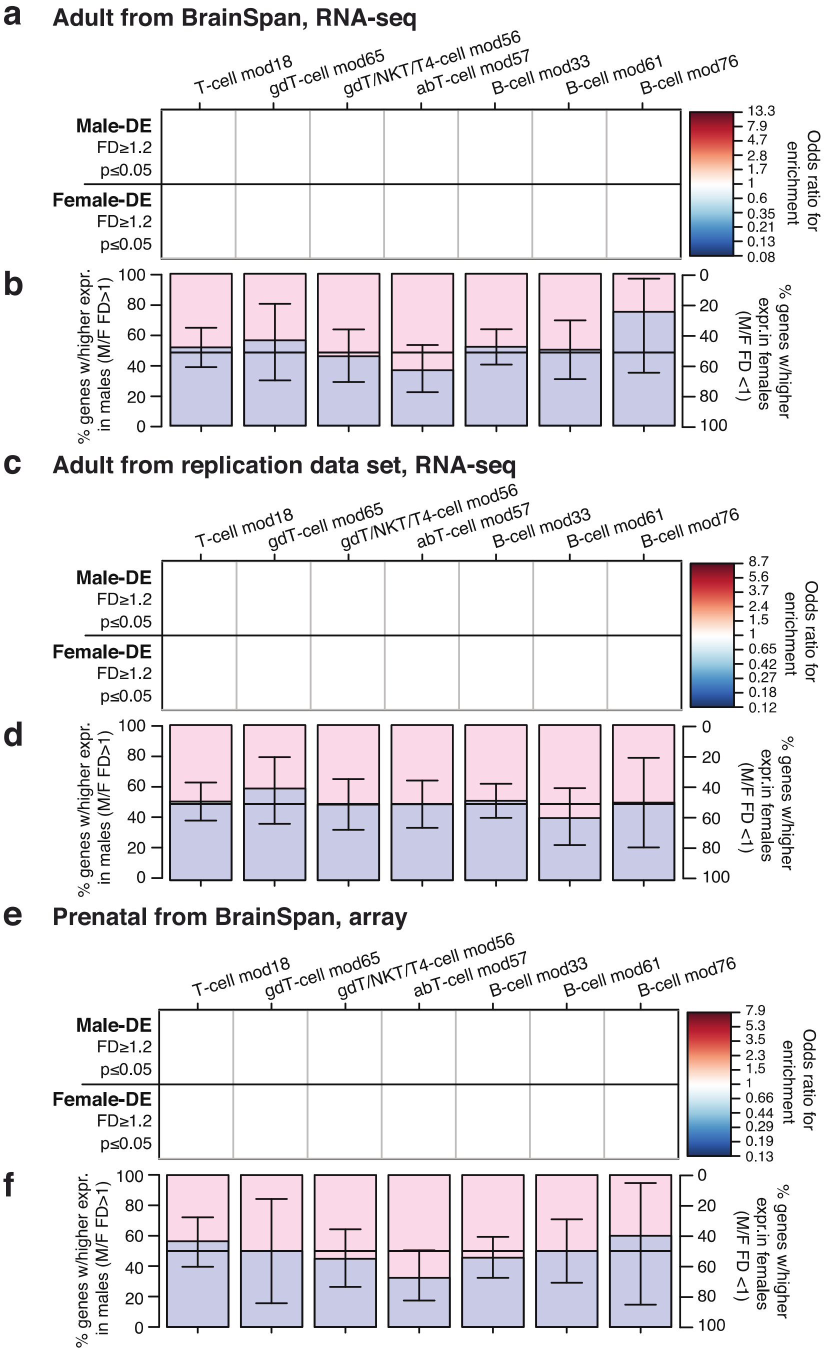

Supplementary Figure 1: Enrichment and sex-differential expression skew for gene sets marking T- and B-cells. (**a**,**c**,**d**) Enrichment for co-expression modules associated with T- and B-cell lineage regulators within male-DE and female-DE gene sets by Fisher's exact test from (**a**) adult samples from BrainSpan (439 male-DE and 427 female-DE genes), (**c**) adult samples from the replication set (268 male-DE and 251 female-DE genes), and (**e**) prenatal samples (509 male-DE and 528 female-DE genes); circle size and color indicate the odds ratio of all significant overlaps (Bonferroni-adjusted p-value <0.05), overlaid text displays the adjusted p-value for each enrichment. (**b**,**d**,**f**) Shifts in the distribution of sex-differential expression direction for genes in each T- and B-cell-associated module in (**b**) adult samples from BrainSpan, (**d**) adult samples from the replication set, and (**f**) prenatal samples from BrainSpan. Blue and pink bars display the proportions of each gene set that have higher expression in males (FD>1) or females (FD<1); whiskers note 95% confidence intervals; horizontal black lines note the proportion of male- and female-higher genes in the corresponding background gene set. Overlaid text displays significant Bonferroni-adjusted p-values from the binomial test. gdT-cell, gamma delta T-cell; NKT, natural killer T-cell; abT, alpha beta T-cell.

Supplementary Figure-2

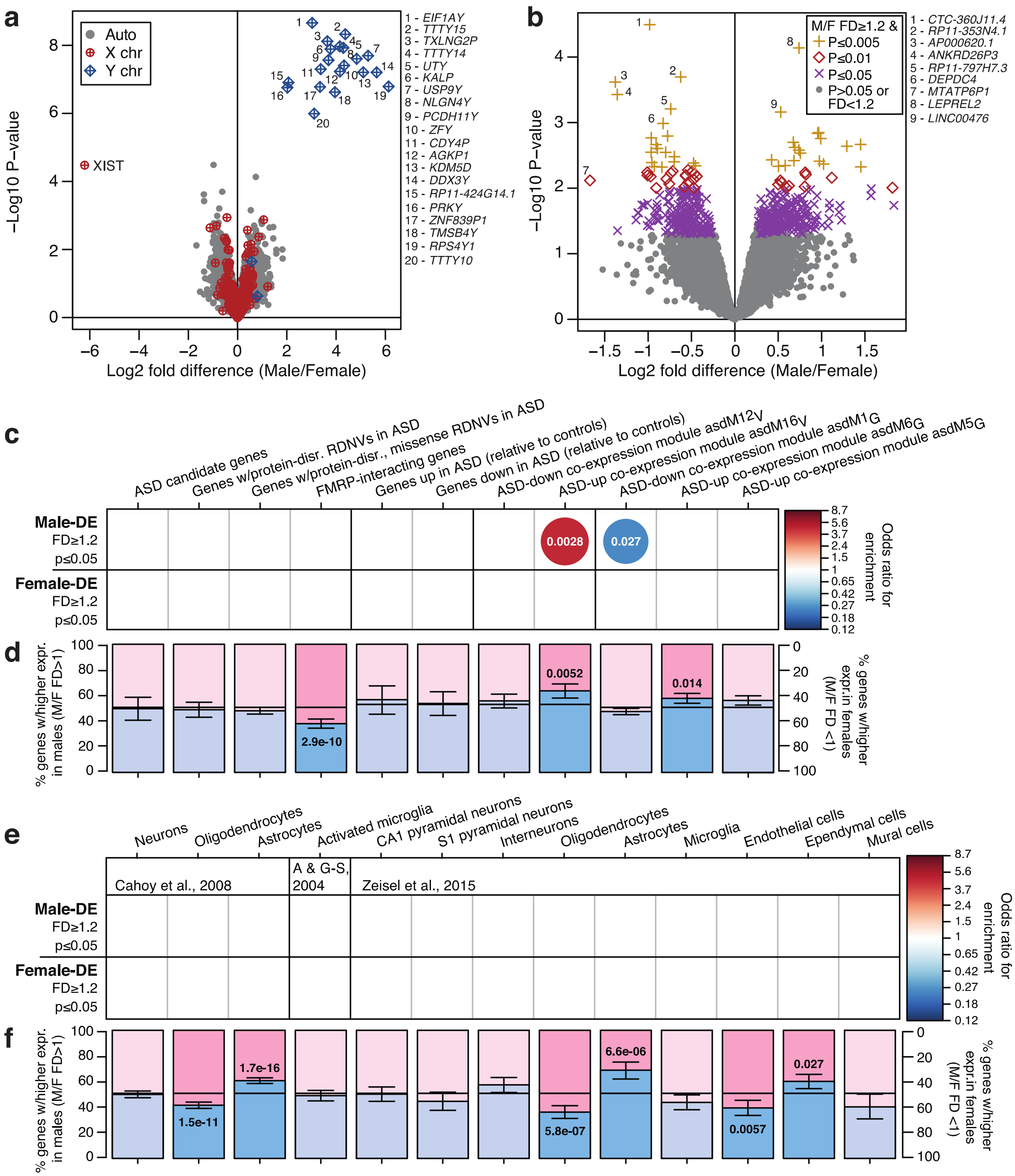

Supplementary Figure 2: Sex-differential gene expression in adult cortex from an independent, replication sample. (**a**) Volcano plot for all 19,354 transcripts expressed in the replication sample (n male=7 samples from 5 subjects; n female=6 samples from 5 subjects). (**b**) Subset of the plot in **a** for the 18,676 autosomal transcripts. (**c**) Enrichment for ASD risk genes and ASD-associated gene expression patterns, and (**e**) neural cell type markers, within male-DE (268 genes) and female-DE (251 genes) gene sets by Fisher's exact test; circle size and color indicate the odds ratio of all significant overlaps (Bonferroni-adjusted p-value <0.05), overlaid text displays the adjusted p-value for each enrichment. (**d**) Shifts in the distribution of sex-differential expression direction for genes in each ASD risk or ASD expression sets, and (**f**) neural cell type markers. Blue and pink bars display the proportions of each gene set that have higher expression in males (FD>1) or females (FD<1); whiskers note 95% confidence intervals; horizontal black lines note the proportion of male- and female-higher genes in the corresponding background gene set. Overlaid text displays significant Bonferroni-adjusted p-values from the binomial test. M, Male; F, Female; RDNVs, rare de novo variants.

Supplementary Table 1: Sample information for adult and prenatal data sets

Data set	Subject	Sex	Age (years or PCW)	Ethn.	Hemi.	Site/Brain bank	pH	PMI (hours)	N cortex samples
BrainSpan, Adult	HSB124	F	13	A	R	Yale	6.34	19.5	3
	HSB119	M	15	A	L	USC	6.93	14.5	3
	HSB105	M	18	E	L	USC	6.21	28	1
	HSB127	F	19	E	L	Yale	5.91	9.5	3
	HSB130	F	21	E	L	Yale	6.81	18	10
	HSB136	M	23	A	R	USC	6.36	10.5	9
	HSB126	F	30	E	R	Yale	6.92	9.5	8
	HSB145	M	36	E	R	Yale	NK, imp. 6.52	18	9
	HSB123	M	37	A	R	Yale	6.37	13	7
	HSB135	F	40	A	R	USC	6.82	30.5	5
Replication set, Adult	UMB5168	F	16	NK	NK	NICHD-BTB	NK	NK (imp 23.17)	1
	AN17425	M	16	NK	NK	Harvard-ATP	NK	26.16	2
	AN19760	M	28	NK	NK	Harvard-ATP	NK	23.25	1
	AN15566	F	32	NK	NK	Harvard-ATP	NK	28.92	1
	UMB5079	M	33	NK	NK	NICHD-BTB	NK	NK (imp 23.17)	2
	AN08161	F	36	NK	NK	Harvard-ATP	NK	23.83	2
	AN10679	F	41	NK	NK	Harvard-ATP	NK	14	1
	AN01410	M	41	NK	NK	Harvard-ATP	NK	27.17	1
	AN15088	F	52	NK	NK	Harvard-ATP	NK	17.88	1
	AN01125	M	56	NK	NK	Harvard-ATP	NK	24.17	1
BrainSpan, Prenatal	HSB154	M	16	A/E	R	NA	6.44	3	7
	HSB96	M	16	H	L&R	NA	6.51	2	9
	HSB97	F	17	E	R	NA	6.51	1	7
	HSB100	F	19	A	L&R	NA	6.56	4	9
	HSB107	F	21	E	L&R	NA	6.61	20	18
	HSB99	F	21	As	L&R	NA	6.51	2	9
	HSB92	M	21	A	R	NA	6.65	4	9
	HSB159	M	22	E	L&R	NA	6.58	2	18

M, Male; F, Female; PCW, post-conception weeks; PMI, post-mortem interval; L, left; R, right; A,African American; E, European; H, Hispanic; As, Asian; NK, not known; imp, imputed.
